# Health-Related Internet Use in Hard-to-Reach Populations: Empirical Findings From a Survey in a Remote and Mountainous Province in China

**DOI:** 10.2196/12693

**Published:** 2019-05-31

**Authors:** HongMin Li, Jin Xu, Lingui Li, Qingyue Meng

**Affiliations:** 1 National Health Commission Key Laboratory of Health Economics and Policy Research School of Health Care Management Shandong University Ji'nan China; 2 China Center for Health Development Studies Peking University Beijing China; 3 School of Public Health Peking University Beijing China; 4 Zhejiang Chinese Medical Univeristy Hangzhou China

**Keywords:** eHealth, rural population, cross-sectional survey, China

## Abstract

**Background:**

The expanding use of the internet contributes to more effective searches for health-related information and opens up opportunities for direct Web-based communication with health care professionals. However, little is known about how users’ characteristics on the demand side influence health-related internet use, especially in remote and rural areas within developing countries. The absence of accurate estimates of users’ characteristics and their impact on adaptations of health care services in developing countries constrains focused policy-centered discussions and the design of appropriate policies.

**Objective:**

The aim of this study was to assess the prevalence of health-related internet use and to identify its determinants in a remote province in China.

**Methods:**

We conducted a cross-sectional survey in June and July of 2018 in Ningxia, located in northwestern China. Rural households were selected by multistage random sampling, and households’ key members were interviewed face-to-face at the respondents’ home. Dependent variables were whether the households use Web health services or not. Independent variables were chosen based on the Andersen behavioral model. Sociodemographic characteristics were compared between households that used health-related Web services with nonusers. We applied logistic regression models to evaluate multivariate associations between respondents’ characteristics and their usage of Web-based health services and obtained odds ratios with 95% CI.

**Results:**

A total of 1354 respondents from rural households were interviewed, of whom 707 (52.22%) were men. The mean age of the respondents was 44.54 years (SD 10.22). Almost half of the surveyed households (640/1354, 47.27%) reported using 1 or more Web-based health care services, whereas 37.8% (502/1354) reported using the internet to obtain health-related information, 15.51% (210/1354) used the internet to communicate with professionals about health issues, and 7.24% (98/1354) had engaged in Web-based consultations in the last year. After controlling for potential confounders, households engaged in health-related internet use were found to be wealthier, have higher health demands, and have less geographic access to high-quality health care compared with other households.

**Conclusions:**

The internet has become a major health information resource in rural Ningxia. Social structures, family enabling factors, health needs, and characteristics relating to health care access were significant predictors of households’ health-related internet use in rural and remote areas in China. Those who belong to older age groups, have low income, and whose education levels do not extend beyond primary school education are significantly less likely to use Web-based health care services and to benefit from Web-based health care programs. A need for continued collaborative efforts involving multiple stakeholders, including communities, Web-based and other health care providers, family members, and the government is needed.

## Introduction

### Key Challenges in China’s Health Care System

The progress of the health care reform, initiated in China in 2009, has led to significant changes in the country’s health care system. China’s achievements in strengthening its health care system have been remarkable, as evidenced by the substantial scale of inputs, efforts, and social expectations [1]. Nevertheless, there are challenges in the accessibility and quality of health care and the equitable allocation of associated resources. A particular challenge is the disparity in health care across regions, rural and urban areas, and different segments of the population [2]. These disparities, including inequitable and inadequate allocation of resources, critically impact the health care system and constitute major barriers that affect health care accessibility in rural and remote areas [3].

The health service delivery system in China is divided into rural and urban parts. The urban part is set up with community-level services as the grassroots level, with collaboration between community health institutions and city hospitals. The rural health system is set up with county-level hospitals as the backbone, with township- and country-level clinics as the grassroots level. At higher levels, large general hospitals at the city, provincial, and national levels provide acute and emergency care and focus on severe and complicated diseases. However, the current health resource allocation is mostly concentrated on large general hospitals, which leads to disparities between the urban and rural areas [4]. Geographically, disparities still exist among eastern, central, and western China [2].

The rural areas in western China have special geography and economy characteristics; therefore, those residents have poor access to health services and are hard-to-reach populations. “Hard-to-reach” is a term used to describe populations that are difficult to reach or involve in research or public health programs because of their physical and geographical location (eg, in mountains, forests, or deserts) or their social and economic situation [5].

### The Internet and Health Care Accessibility

Expansion of internet use contributes to more effective searches for health-related information and creates opportunities for direct communication with health care professionals through the internet. Health-related internet use can potentially reduce overall medical costs [6] by eliminating the need for diagnostic testing as well as travel expenses incurred by rural patients who repeatedly visit urban hospitals. Moreover, by encouraging users to have regular medical checkups, Web-based health care services may lead to a reduction in the number of emergency cases [6]. Electronic health (eHealth), defined as the use of emerging information and communications technology (ICT), and especially the internet for health-related issues, has become an important supplement to traditional health care resources [7].

Health care can be delivered to geographically remote locations via the internet, which can potentially play an important role in facilitating access to medical information and service delivery in rural and remote areas for hard-to-reach populations. The internet provides access to quality health care for those who may be underserved by the traditional medical system [8,9], thus addressing disparities in health care [10]. Moreover, studies have shown that internet-based health care training, education, and research programs targeting health care providers in rural and remote areas have led to increased communication among clinicians, resulting in more efficient, higher quality, and less costly care in these areas [11,12].

### The Internet and Health Care in China

With the rapid development of the internet and ICT in China, 802 million people were reported to have access to the internet in 2018, of which 26.3% were from rural areas [13]. The government is actively promoting integrated provision of internet and health care facilities. The Internet Plus health care strategy was proposed in 2015 by the National Development and Reform Commission and the National Health and Family Planning Commission (renamed the National Health Commission) to improve access to health care in rural and remote areas through ICT. Extensive investments of social capital have been made in parallel in Web-based health services available on the internet through the development of consultations, hospital facilities, and medicines as well as in health management. However, the effects and consequences of large investments of capital in the emerging Web-based health care market on the performance and accessibility of health care remain unclear.

Disparities in health care across geographical, socioeconomic, racial, and ethnic groups can only be reduced through effective eHealth designs and practices that are responsive to public demands [10]. Evidence of the demanding characteristics and determinants are needed to improve efficiency and effectiveness of policies and investments. However, the limited availability of demand-related information has resulted in uncertainty regarding the performance of eHealth programs and their adoption.

### Research on Electronic Health Usage

Previous studies [14-17] on the internet and health care, which have mainly focused on developed countries, have contributed to a better understanding of their characteristics and impacts on adaptations of health care services. They have shown that people who use the internet for health-related purposes are more likely to be young and female, with high levels of income and education. Moreover, factors such as health status and attitudes toward the internet have been found to influence Web health-related activities.

As per the World Health Organization, in a low-resource setting, ICT can be a key component for providing universal health coverage [18]. However, studies conducted in developing countries [19-22] have only focused on internet hospitals, the market for Web-based consultations, and the government’s role in the provision of eHealth. Their findings were based on secondary data and Web-based resources. In contrast, less is known about how users’ characteristics on the demand side influence health-related internet use, especially for hard-to-reach populations living in remote areas in developing countries. The absence of accurate estimates of users’ characteristics and their impact on adaptation of health care services in developing countries limits focused policy-centered discussions and the design of appropriate policies.

For populations facing barriers to health care access, the internet can be a particularly appealing source of health information and professional advice. It is important to know how internet-based health care services have been used among such hard-to-reach populations. Previous studies were mostly conducted in cities, and a few studies were conducted among hard-to-reach populations. Furthermore, previous studies lacked a concept framework in their methods.

### Objectives

The aims of this study were to assess the prevalence of health-related internet use, to identify its determinants in rural areas in a western province in China, and to make policy recommendations for Web-based health care development in hard-to-reach populations. We sought to answer the following research questions: (1) What are the characteristics of users of Web-based health care services in remote areas? (2) What are the determinants of health-related internet use among the hard-to-reach populations?

## Methods

### Study Site

Ningxia, located in northwestern China, is the representative region for remote areas in China. Of the 6.68 million inhabitants of Ningxia, 45% were living in rural areas at the end of 2016. In 2016, the number of medical and technical personnel per 1000 inhabitants was 3.9 in rural Ningxia, compared to the national number of 6.1. Furthermore, the number of beds was 3.33 per 1000 people in this region compared to the national number of 5.37 [23]. In addition, Ningxia has a variety of complex landforms—mountains, plateaus, plains, hills, and valleys—that affect the population’s geographic accessibility to health care, especially in rural areas.

### Study Design and Data

We conducted a cross-sectional survey in June and July 2018 in Ningxia. Of the 22 counties and districts in Ningxia, 4 were selected by considering the socioeconomic factors and environmental and health care resources (Table 1). Of the selected counties and districts, 46 rural villages were chosen for the survey. Within each village, about 30 households were randomly selected. The respondents within the selected households had to meet the following inclusion criteria: age of 18-65 years and possessing the right to make decisions related to the use of health care services within their families. Only one respondent in a family was selected and responsible for the family’s information on the Web-based health-related service usage. Face-to-face interviews were conducted at the respondents’ homes, and the mean time required for completing the survey was around 30 min.

### Measurements

The Andersen Behavioral Model of Health Services Use is a conceptual framework widely used to investigate demands for health care [24]. The model enhances our understanding of the reasons individuals use health services and promotes equitable access to health care services. It proposes determinants to explain why people use health care services, such as an individual’s predisposition to use services, factors that enable or impede use, and people’s need for care. Even if the model has been widely applied to the general health service field, few studies apply it to the Web health care context [25]. Web-based health care is an innovation used in health care services; this model can help develop a comprehensive adoption model and explain why people use the internet to obtain health information and services.

The dependent variables in our analysis were the respondents’ internet search for health-related information, Web-based communication with health professionals, and Web-based consultation. A Web-based consultation takes place on professional medical platforms or websites, and the process of consultation includes history taking, diagnosis, and intervention advice. Web-based communication is more casual: It can occur on social apps between patients and professionals, and the context of communication is health related. Use of the Web-based health care services provided was judged based on whether the respondents use any of the three abovementioned services. Table 2 presents the definitions and response options for the selected variables.

**Table 1:** Economic development and human resources for health in the selected counties or districts.

Counties/districts	Disposable income (Renminbi, in rural areas)	Number of doctors per 1000 people
Tongxin	6710.69	0.84
Xiji	6857.12	1.16
Huinong	10995.46	3.12
Qingtongxia	11200.00	1.24
Ningxia	9118.69	2.14

**Table 2:** Definitions and response options for the selected dependent and independent variables.

Variables			Options
**Dependent variables**			
	Use of Web-based health care services^a^		Yes/no
	Searching the internet for health-related information^a^		Yes/no
	Web-based communication with health professionals^a,b^		Yes/no
	Web-based consultation^a,b^		Yes/no
**Independent variables**			
	**Social structure factors**		
		Education levels	No formal education, primary school, junior middle school, high school, college and above
		Occupation	Farmer, informally or formally employed
		Ethnic groups	Han, Hui, other
	**Family enabling factors**		
		Household annual income	Renminbi
		Health knowledge scores^c^	Range: 1-5
	**Health care accessibility factors**		
		Distances to county hospital	Kilometers
		Distances to city hospital	Kilometers
	**Health need characteristics**		
		Does the household have chronic patient(s)?^d^	Yes/no
		Did the household have outpatient(s) in the last month?	Yes/no
		Did the household have inpatient(s) in last 12 months?	Yes/no
		Does the household have older persons (age≥60 years)?	Yes/no
		Does the household have kids (age≤5 years)?	Yes/no
	**Other confounding factors**		
		Gender	Female/male
		Age	Number of years
		Family members	Number

^a^Internet use for health purposes was measured as usage by all family members for addressing their health issues over the last 12 months.

^b^A Web-based consultation must include history taking, diagnosis, and intervention advice and can take place on the internet platforms of professional medical services. Web-based communication is more casual; it can occur on social apps between patients and professionals, and the context of communication is health related.

^c^Five questions on health-related knowledge were posed, entailing true/false responses.

^d^Chronic diseases were hypertension, dyslipidemia, diabetes or high blood sugar, cancer or malignant tumor, cardiovascular disease, stroke, chronic lung diseases, liver disease, stomach or other digestive disease, arthritis or rheumatism, asthma, emotional or psychiatric problems, and memory-related disease.

### Data Analysis

We first performed a descriptive analysis to obtain a basic sociodemographic profile of respondents and their characteristics. The sociodemographic characteristics of households that used Web-based health-related services were then compared with those that did not use these services. We performed a univariate analysis for each of the variables considered by using an appropriate statistical test (Chi-square test for discrete variables and *t* test for continuous variables).

We applied logistic regression models to evaluate multivariate associations between respondents’ characteristics and their usage of Web-based health services and obtained odds ratios (ORs) with 95% CIs. Regression analysis was performed according to the following equation:

Pr[y_i_ = 1|SS, FE, HA, HN, O] = F(β_0_ + β_1_SS_i_ + β_2_FE_i_ + β_3_HA_i_ + β_4_HN_i_ + β_5_O_i_)

Where y is an indicator of health-related internet use; SS, FE, HA, HN, and O are measures of social structure, family enabling factors, health care accessibility, health care need, and other confounding factors, respectively; and F denotes the logistic cumulative distribution function.

Regression analysis was performed on the sample as a whole and for each individual Web-based health-related activity, namely, searches conducted for health-related information, communication with professionals, and Web-based consultations. In addition, we performed regression analysis for households that availed of any of these three Web-based activities to obtain health care assistance. A *P* value <.05 was considered statistically significant. Stata version 14.0 for Microsoft Windows (Stata Corp, College Station, TX) was used for the statistical analysis.

## Results

### Study Population

Descriptive statistics for the study sample are presented in Table 3. A total of 1354 respondents from rural households were interviewed, of whom 707 (52.22%) were men. The mean age of the respondents was 44.54 years (SD 10.22). More than three-quarters of the respondents (1038/1354, 76.66%) were able to use the internet. In this study, those who were able to use the internet were considered to be able to use social apps such as WeChat to contact with people or search information on their mobile phone or computer with an internet connection.

An examination of the factors related to the social structure revealed that more than 74.15% (1004/1354) of the respondents were farmers, most of whom lacked an education beyond junior middle school (1176/1354, 86.86%). The majority of the respondents (917/1354, 67.73%) were of Han ethnicity. In 2017, the mean household annual income was ¥39,150.17 (or US $5725.63), and the mean score for health-related knowledge was 3.57 (range: 0-5). Almost half of the selected households (619/1354, 45.72%) had members who were chronic patients, 37.81% (512/1354) of households had used outpatient services in the last 1 month, and 35.86% (486/1354) of households had used inpatient services in the last 12 months. The mean distances of the surveyed households from county- and city-based hospitals were 21.39 km (SD 30.04) and 69.19 km (SD 42.74), respectively.

### Overall Health-Related Internet Use

Almost half of the surveyed households (640/1354, 47.27%) reported using one or more Web-based health care services, 37.8% (502/1354) reported using the internet to obtain health-related information, 15.51% (210/1354) used the internet to communicate with professionals about health issues, and 7.24% (98/1354) had engaged in Web-based consultations in the last year (Table 4).

We conducted a univariate analysis of differences among households that had used and those that had not used Web-based services for all of the variables. As shown in Table 2, the overall utilization of Web-based services was associated with the respondents’ positions within the social structure, family enabling factors, family members’ health status, and geographic access to health care. Specifically, respondents with the following characteristics were more likely to use Web-based health care services: higher education levels, higher health-related knowledge scores (3.79 vs 3.57), higher household incomes (¥50,514 vs ¥28,963 or US $7399 vs US $4242), higher health demands, and living nearer to county- or city-based hospitals (Table 5).

### Determinants of Health-Related Internet Use

Table 6 shows the determinants of health-related internet use. The ORs from the logistic regression analysis were summarized after controlling for potential confounders. For the category of factors related to the social structure, households with higher levels of education and formal or informal jobs were more likely to use Web-based health services. There was no difference in the use of Web-based health services among respondents of Han and Hui ethnicities. For the category of family enabling factors, respondents with higher health-related knowledge scores were more likely to seek Web-based communication (OR 1.43) or Web-based consultations (OR 1.40) with health care professionals. High-income households were more likely to search for health-related information (OR 1.25) and communicate through the internet on health issues (OR 1.44). Findings for the health need characteristics revealed that households with higher health demands were more likely to use Web-based health services. In particular, households that included outpatients over the last 1 month were more likely to search for Web-based health-related information (OR 1.38), perform Web-based communication (OR 1.99), and schedule Web-based consultation (OR 2.00). Findings on the geographic factors related to health care access revealed that greater distances to city-based hospitals were more likely to prompt members of households to search for health-related information (OR 1.27), perform Web-based communication with professionals (OR 1.60), and schedule Web-based consultations (OR 1.80). Younger respondents were more likely to opt for health-related internet use than older respondents.

**Table 3:** Characteristics of the study sample (N=1354).

Variable				Value
**Social structure, n (%)**				
	**Education level**			
		No formal education	306 (22.60)	
		Primary school	403 (29.76)	
		Junior school	472 (34.86)	
		High school	116 (8.57)	
		College and above	57 (4.20)	
	**Occupation**			
		Farmers	1004 (74.15)	
		Others	350 (25.85)	
	**Ethnic groups**			
		Han	917 (67.73)	
		Hui	437 (32.27)	
**Family enabling factors, mean (SD)**				
	Health knowledge			3.57 (1.18)
	Family income (Renminbi)			39,150.17 (51,196.3)
**Health need factors, n (%)**				
	**Members of age <5 years**			
		Yes	394 (29.10)	
		No	960 (70.90)	
	**Members of age >60 years**			
		Yes	406 (29.99)	
		No	960 (70.90)	
	**Members with chronic conditions**			
		Yes	619 (45.72)	
		No	735 (54.28)	
	**Outpatient clinic visit in last 1 month**			
		Yes	512 (37.81)	
		No	842 (62.19)	
	**Hospitalization in last 12 months**			
		Yes	486 (35.89)	
		No	868 (64.11)	
**Health accessibility, mean (SD)**				
	Distance to county hospital (km)			21.39 (30.04)
	Distance to city hospital (km)			69.19 (42.74)
**Gender, n (%)**				
	Female			647 (47.78)
	Male			707 (52.22)
Age (years), mean (SD)				44.54 (10.22)
Family size, mean (SD)				4.43 (1.60)

**Table 4:** The prevalence of health-related internet use (N=1354).

Variables	n (%)
Use of Web-based health care services	640 (47.27)
Searching internet for health-related information	502 (37.8)
Web-based communication with health professionals	210 (15.51)
Web-based consultation	98 (7.24)

**Table 5:** Characteristics of the study sample in relation to Web-based health-related activities.

Indicators/variables			Overall use of Web-based services (n=640)	*P* value	Web-based information use (n=502)	*P* value	Web-based communication (n=210)	*P* value	Web-based consultation (n=98)	*P* value
**Social structure**										
	**Education level, n (%)^a^**			<.001		<.001		<.001		<.001
		No formal education	66 (21.57 )		34 (11.11)		16 (5.23)		6 (1.96)	
		Primary school	161 (39.95)		112 (27.79 )		54 (13.40)		20 (4.96)	
		Junior school	278 (58.90)		232 (49.15)		79 (16.74)		38 (8.05)	
		High school	84 (72.41 )		74 (63.79)		33 (28.45)		17 (14.66)	
		College and above	51 (89.47)		50 (87.72)		28 (49.12)		17 (29.82)	
	**Occupation, n (%)^a^**			<.001		<.001		<.001		<.001
		Farming	407 (40.54)		302 (30.08)		111 (11.06)		48 (4.78)	
		Others	233 (66.57)		200 (57.14)		99 (28.29)		50 (14.29)	
	**Ethnic groups, n (%)^a^**			.94		.54		.02		.09
		Han	434 (47.33)		345 (37.62)		128 (13.96)		59 (6.43)	
		Hui	206 (47.14)		157 (35.93)		82 (18.76)		39 (8.92)	
**Family enabling factors, mean (SD)**										
	Health knowledge score^b^		3.79 (1.17)	<.001	3.84 (1.17)	<.001	4.05 (1.10)	<.001	4.12 (1.04)	<.001
	Family income (Renminbi)^b^		50514.77 (64409.1)	<.001	53223.2 (67844.9)	<.001	57890.55 (43781.19)	<.001	55173.62 (59225.8)	.001
**Health need factors, n (%)**										
	**Members of age <5 years^a^**			.19		.03		.07		.02
		Yes	197 (50)		163 (41.37)		72 (18.24)		38 (9.64)	
		No	443 (46.15)		339 (35.31)		138 (14.37)		60 (6.25)	
	**Members of age >60 years^a^**			.54		.58		.86		.08
		Yes	197 (48.52)		155 (38.18)		64 (15.76)		37 (9.11)
		No	443 (46.73)		347 (36.6)		146 (15.40)		61 (6.43)	
	**Members with chronic conditions^a^**			.01		.46		.13		.08
		Yes	315 (50.89)		236 (38.13)		106 (17.12)		53 (8.56)
		No	325 (44.22)		266 (36.19)		104 (14.15)		45 (6.12)	
	**Outpatient clinic visit in last 1 month^a^**			<.001		.02		<.001		.001
		Yes	276 (53.91)		209 (40.82)		108 (21.09)		53 (10.35)
		No	364 (43.23)		293 (34.80)		102 (12.11)		45 (5.34)	
	**Hospitalization in last 12 months^a^**			<.001		.08		.004		.05
		Yes	263 (54.12)		195 (40.12)		94 (19.34)		44 (9.05)	
		No	377 (43.43)		307 (33.37)		116 (13.36)		54 (6.22)	
**Health accessibility**										
	Distance to county hospital (km), mean (SD)^b^		17.42 (18.13)	<.001	17.54 (19.62)	<.001	18.06 (30.04)	.08	19.37 (26.68)	.49
	Distance to city hospital (km), mean (SD)^b^		65.22 (44.34)	.001	64.09 (43.62)	<.001	72.83 (47.47)	.17	76.27 (44.35)	.04
**Gender^a^**				.89		.66		.06		.31
	Female, n (%)		307 (47.45)		236 (36.48)		88 (13.60)		42 (6.49 )	
	Male, n (%)		333 (47.10)		266 (37.62)		122 (17.26 )		56 (7.92)	
Age (years), mean (SD)^b^			42.22 (10.56)	<.001	41.03 (10.47)	<.001	41.01 (10.88)	<.001	39.03 (11.41)	<.001
Family size, mean (SD)^b^			4.43 (1.56)	.89	4.42 (1.66)	.87	4.52 (1.49)	.33	4.41 (1.60)	.23

^a^Pearson Chi-squared test was performed for the univariable analysis.

^b^*t* test was performed.

**Table 6:** Multivariate associations of respondents’ and households’ characteristics and Web-based health-related activities.

Variables				Web-based service use (n=640), OR^a^ (95% CI)		*P* value		Web-based information use (n=502), OR (95% CI)		*P* value		Web-based communication (n=210), OR (95% CI)		*P* value		Web-based consultation (n=98), OR (95% CI)		*P* value	
**Social structure**																			
		**Education (reference: no formal education)^a^**																	
			Primary school		2.16 (1.51-3.10)		<.001		2.55 (1.65-3.94)		<.001		2.41 (1.32-4.42)		.004		2.19 (0.84-5.65)		.10
			Junior school		4.05 (2.79-5.88)		<.001		5.55 (3.59-8.58)		<.001		2.72 (1.47-5.02)		.001		3.32 (1.31-8.43)		.01
			High school		6.67 (3.88-11.48)		<.001		9.18 (5.21-16.18)		<.001		4.25 (2.07-8.71)		<.001		5.11 (1.80-14.49)		.002
			College and above		15.26 (5.87-39.64)		<.001		25.90 (10.20-65.74)		<.001		8.27 (3.53-19.35)		<.001		9.89 (3.15-30.99)		<.001
	**Occupation (reference: farming)^b^**																		
			Employed		1.27 (0.93-1.74)		.12		1.19 (0.87-1.64)		.25		1.72 (1.17-2.52)		.006		1.71 (1.01-2.90)		.04
	**Ethnic group (reference: Han)^b^**																		
			Hui		0.83 (0.61-1.15)		.27		0.75 (0.53-1.04)		.09		1.21 (0.80-1.84)		.36		0.97 (0.54-1.74)		.93
**Health need factors**																			
	**Members of age <5 years (reference: No)^b^**																		
			Yes		0.91 (0.67-1.22)		.54		0.97 (0.71-1.33)		.86		0.97 (0.66-1.43)		.89		1.11 (0.66-1.86)		.68
	**Members with chronic conditions (reference: No)^b^**																		
			Yes		1.35 (1.05-1.75)		.02		1.19 (0.91-1.56)		.18		1.13 (0.80-1.59)		.47		1.33 (0.84-2.13)		.22
	**Outpatient clinic visit in last 1 month (reference: No)^b^**																		
			Yes		1.57 (1.21-2.04)		.001		1.38 (1.05-1.81)		.01		1.99 (1.42-2.78)		<.001		2.00 (1.27-3.16)		.003
	**Inpatient clinic visit in last 12 months (reference: No)^b^**																		
			Yes		1.63 (1.25-2.12)		<.001		1.30 (0.99-1.71)		.05		1.51 (1.07-2.12)		.01		1.28 (0.80-2.03)		.29
**Family enabling factors**																			
	Family income				1.26 (1.10-1.44)		<.001		1.25 (1.09-1.44)		.002		1.44 (1.18-1.75)		<.001		1.19 (0.938-1.52)		.14
	Health knowledge score				1.13 (1.01-1.26)		.02		1.08 (0.96-1.21)		.19		1.43 (1.22-1.67)		<.001		1.40 (1.12-1.75)		.002
**Health accessibility**																			
	Distance to county hospital				0.61 (0.50-0.77)		<.001		0.68 (0.54-0.85)		.001		0.75 (0.56-1.01)		.06		0.81 (0.54-1.23)		.33
	Distance to city hospital				1.26 (1.01-1.55)		.03		1.27 (1.02-1.58)		.03		1.60 (1.19-2.14)		.002		1.80 (1.18-2.76)		.006
Age				0.97 (0.97-0.99)		.006		0.96 (0.95-0.98)		<.001		1.00 (0.98-1.02)		.74		0.98 (0.96-1.01)		.34	
Family size				0.99 (0.90-1.08)		.76		0.98 (0.90-1.08)		.76		0.98 (0.87-1.10)		.81		0.98 (0.84-1.15)		.86	

^a^OR: odds ratio.

^b^Reference category set to a value of 1.

## Discussion

### Principal Findings

Our findings indicate that in rural and remote areas, almost half (640/1354, 47.27%) of the surveyed households had used the internet to obtain health-related information or Web-based treatment over the last year. Households engaged in health-related internet use were found to be richer, have higher health demands, and have less geographic access to high-quality health care compared with other households.

As previous studies reported [16,17,26,27], the most common way to use the internet for health is to search for health information. The internet has become a major health information resource in rural Ningxia, although the prevalence is less than that in developed countries [16,17,26,27]. At the same time, the internet provides an alternative for households in rural and remote areas to contact professionals about health issues.

Social structures, family enabling factors, health needs, and characteristics related to health care access were significant predictors of households’ health-related internet use in rural and remote areas in China. A more favorable position within the social structure increased the likelihood of Web-based use of health services, as higher education levels or employment potentially increased knowledge and proficiency about internet use as well as eHealth literacy [28]. Family enabling factors, especially household incomes, were found to be an important determinant of Web-based health care use. This finding is consistent with those of other studies [15,17]. As expected, higher household incomes increased the probability of respondents’ use of Web-based health-related services. Higher health demands also increased the likelihood of health-related internet use. Households with either inpatients or outpatients were more likely to use the internet to search for health-related information. Our findings related to the final predictor of health access supported a geographic digital divide, as the likelihood of health-related internet use significantly increased among respondents located at greater distances from city-based hospitals. This finding is consistent with that of a previous study [29], which showed that the use of Web-based health services is associated with limited access to health care. The internet may offer a low-cost source of health information and could help meet the heightened demand for health-related information among those facing barriers of access to care. Before the potential confounders were controlled, people who lived nearer to county- or city-based hospitals were more likely to use Web-based health care services. However, after controlling for potential confounders, the residents who live farther from the city hospitals were found to be more likely to use Web-based health care services, which confirms that residents in low-resource settings are more likely to ask for help from the internet. In addition, residents who lived nearer to the county hospitals were found to be more likely to use Web-based health care services even after controlling for the variables. A possible explanation is that the service provision by county hospitals in western regions is still limited and needs further research and deep exploration.

Our findings also showed that different types of Web-based health care users demonstrate similar as well as contrasting characteristics. Although younger participants were more likely to search for health-related information on the internet, the age factor did not significantly influence Web-based communication with doctors or Web-based consultations. Our findings further indicated that farmers were more likely to search for health-related information on the internet than to conduct other Web-based health-related activities. This finding could be attributed to the fact that farmers have lived in rural areas for a long time and have had few opportunities to work in cities, which could reduce the likelihood of their participation in Web-based communication and consultations.

Although health-related knowledge scores had no significant impact on searches for information on health-related issues, participants whose scores were higher were more likely to conduct Web-based consultations and communication. The presence of extensive health-related information on the internet highlights the importance of health literacy. Health literacy was independently related to health knowledge [30]. Respondents with low levels of health literacy are unable to evaluate health information available on the internet, owing to which, they are misled by unsubstantiated information obtained from the internet [31].

The internet may also play a major role in future health care delivery. Although previous studies [15-17] have examined the characteristics of internet users who engage in Web-based health-related activities, this study is one of the first to examine the characteristics of households within rural and remote areas that include different types of health-related internet users.

In summary, the internet is an important alternative to obtain health information and services for rural and remote residents in China. The findings of this study confirm the results of previous studies [15-17]: Socioeconomically disadvantaged individuals (according to age, income, and education levels) are less likely to access health-related information and engage in Web-based consultations. Those who belong to older age groups, have low incomes, and have education levels less than primary school are significantly less likely to use Web-based health care services and benefit from related health care programs.

### Implications for Policies, Practices, and Future Studies

Our findings indicate a need for continued collaborative efforts involving multiple stakeholders, including communities, Web-based and other health care providers, family members, and the government. Web-based health-related services and programs should focus on the provision of training for older adults, lower-income households, and households located in remote areas to reduce disparities in health care. Health education programs conducted by communities and health care institutions could integrate internet skills development with health education when targeting the abovementioned key population segments.

More specifically, populations should be educated on ways to acquire high-quality and useful health-related information and services from internet. Health knowledge and promotion programs should ensure that education efforts take into account a population’s health literacy skills. Health educators need to be aware of their target population’s health literacy skills and adjust educational interventions accordingly. Considering the characteristics of populations in rural and remote areas, researchers and providers of Web-based health-related services should develop simple and engaging methods for providing information and treatment.

From the family perspective, younger family members and those with higher education levels can provide meaningful assistance for older family members to enhance their health-related internet use. Regulations, legal restrictions, and rules should be clarified and strictly implemented in relation to Web-based consultations and the dissemination of health-related information to strengthen the management of marketing activities associated with Web-based health care delivery.

Further studies should examine the effects of internet usage on the delivery of health care services using longitudinal or experimental research designs and focusing especially on remote and rural areas within developing regions. The association between chronic conditions and the characteristics of specific Web-based health services is another important area of inquiry. Studies should attempt to identify the characteristics of acquired health-related knowledge that may be associated with the likelihood of increased use of health care facilities from the internet, as the conditions of knowledge acquisition may help resolve the barriers to Web-based communication and consultation. Furthermore, intervention studies are needed to examine what works for people with low health literacy skills, to improve their health knowledge and evaluate whether increased knowledge or health literacy results in improved health outcomes.

### Limitations

The findings of this study provide important insights into the use of internet-based health care services among populations located in remote and rural areas and contribute to the evidence base on the delivery of Web-based health care services from the demand perspective. However, there were several limitations to our analysis. First, as this was a cross-sectional study, meaningful differences could only be considered as correlational, not causal. Second, a comprehensive spectrum of health-related Web-based activities such as Web-based purchases of medicines was not considered. Moreover, the study site (northwestern China) is not representative of other regions within the country.

Despite these limitations, these results have significant implications for future research and the development of computer/internet training for inhabitants of rural and remote areas.

### Conclusions

The internet has become a major health information resource in rural Ningxia. Social structures, family enabling factors, health needs, and characteristics related to health care access were significant predictors of households’ health-related internet use in rural and remote areas in China. People who belong to older age groups, have low incomes, and have education levels below primary school are significantly less likely to use Web-based health care services and benefit from Web-based health care programs. There is a need for continued collaborative efforts involving multiple stakeholders, including communities, Web-based and other health care providers, family members, and the government.

## Abbreviations

eHealth: electronic health
ICT: information and communications technology
OR: odds ratio
